# Supplementary material for: Understanding the infection severity and epidemiological characteristics of mpox in the UK
Source: Nat Commun. 2024 Mar 11;15:2199. doi: 10.1038/s41467-024-45110-8 (PMC10928097; doi:10.1038/s41467-024-45110-8)
Supplement: Supplementary file 1 — Supplementary Information [file 41467_2024_45110_MOESM1_ESM.pdf]

Supplementary Tables and Figures

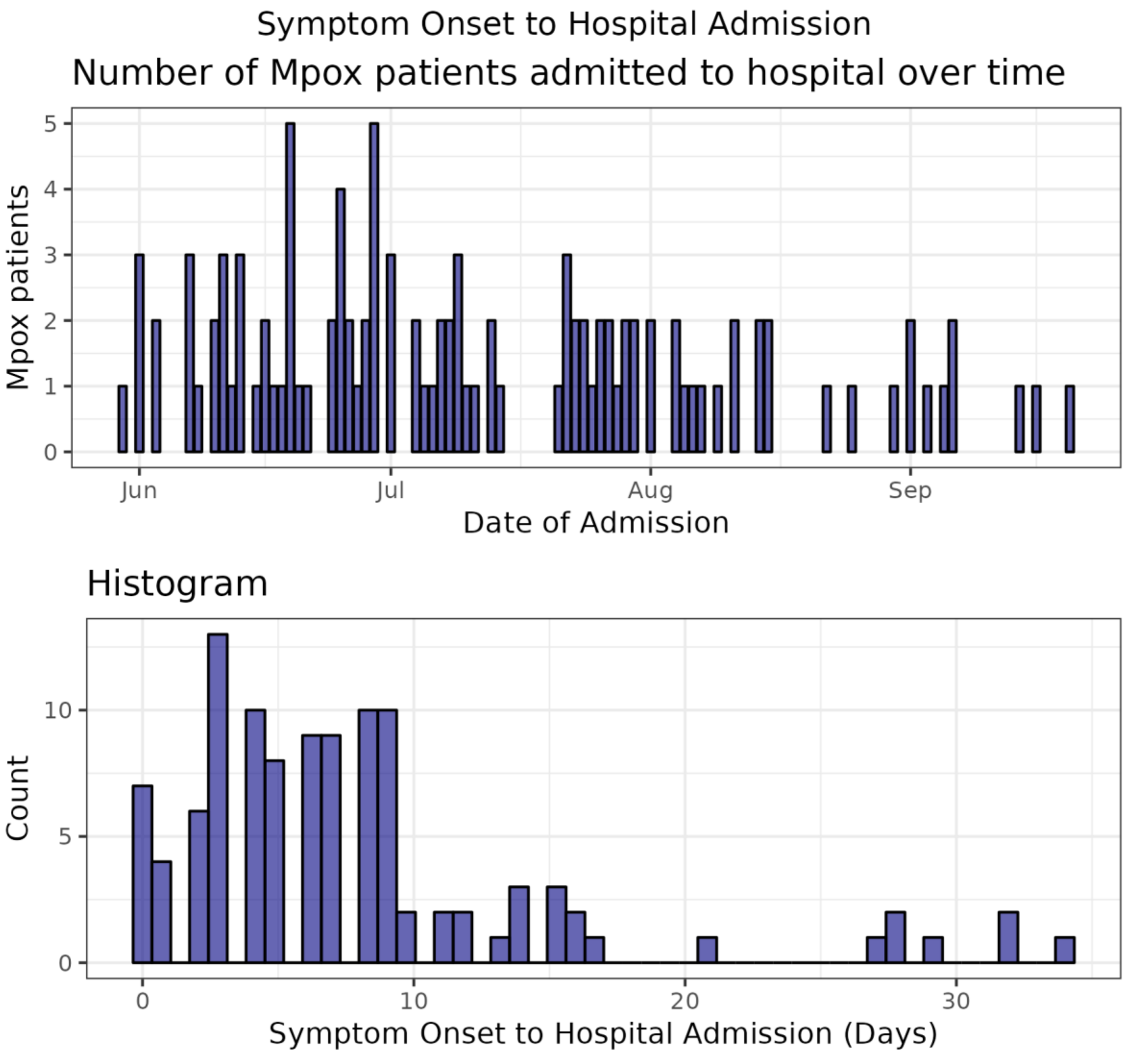

Supplementary Figure 1: The hospital admission dates of patients with an mpox infection that had a symptom onset date and a histogram of the time between symptom onset and hospital admission.

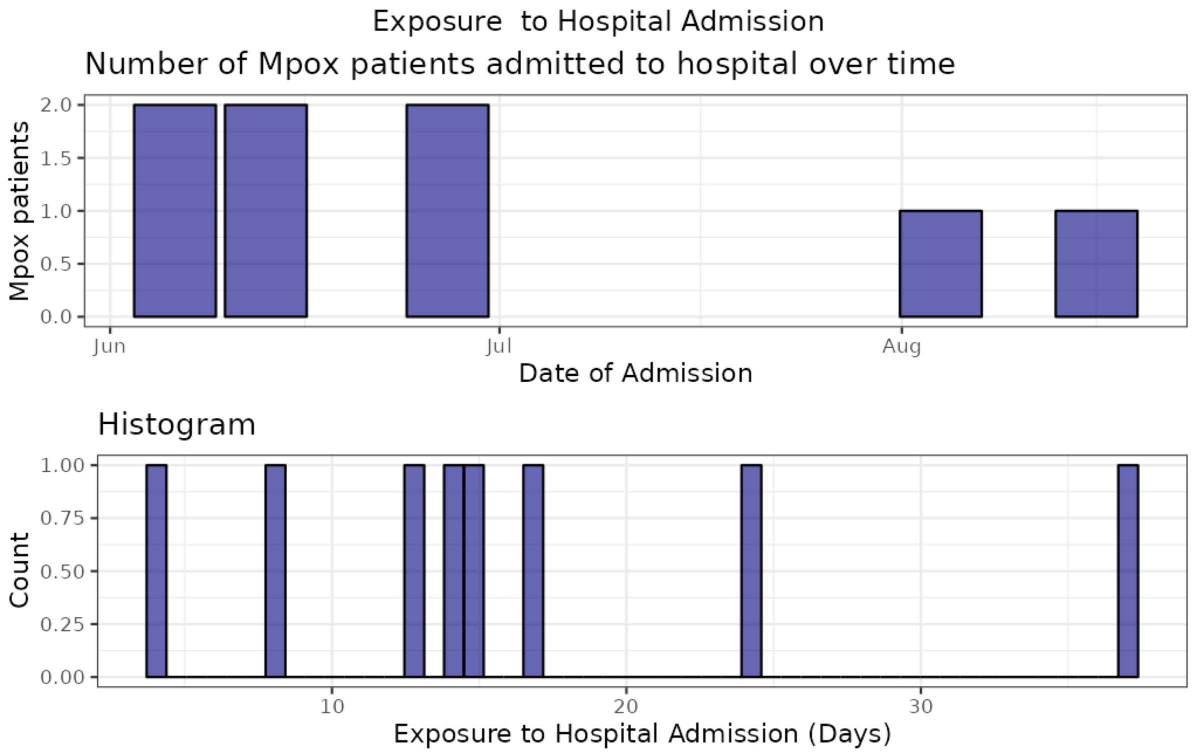

Supplementary Figure 2: The hospital admission dates of patients with an mpox infection that had an exposure date and a histogram of the time between exposure and hospital admission.

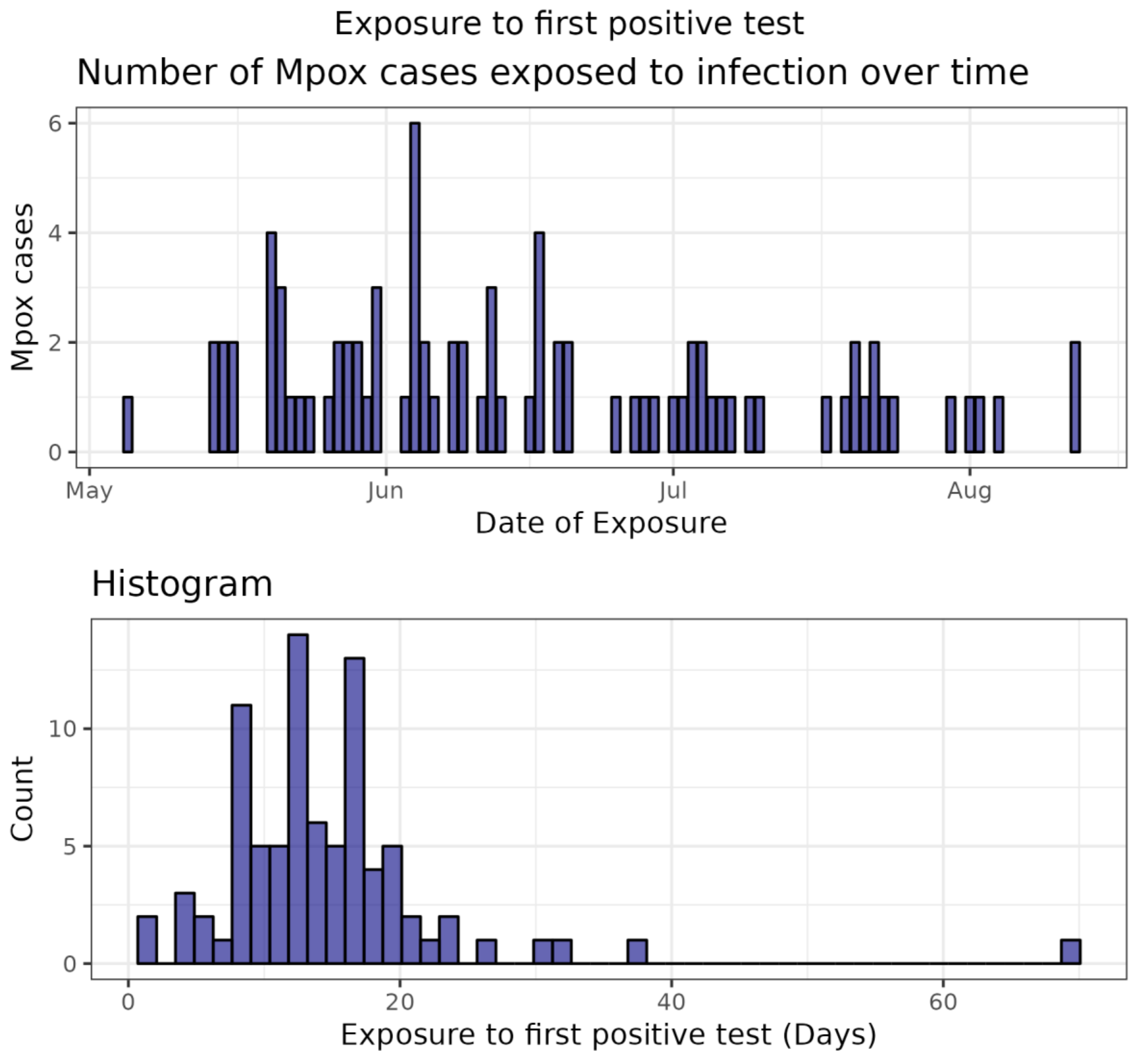

Supplementary Figure 3: The exposure dates of cases with an mpox infection that had both an exposure date and a specimen date, and a histogram of the time between exposure and first positive test.

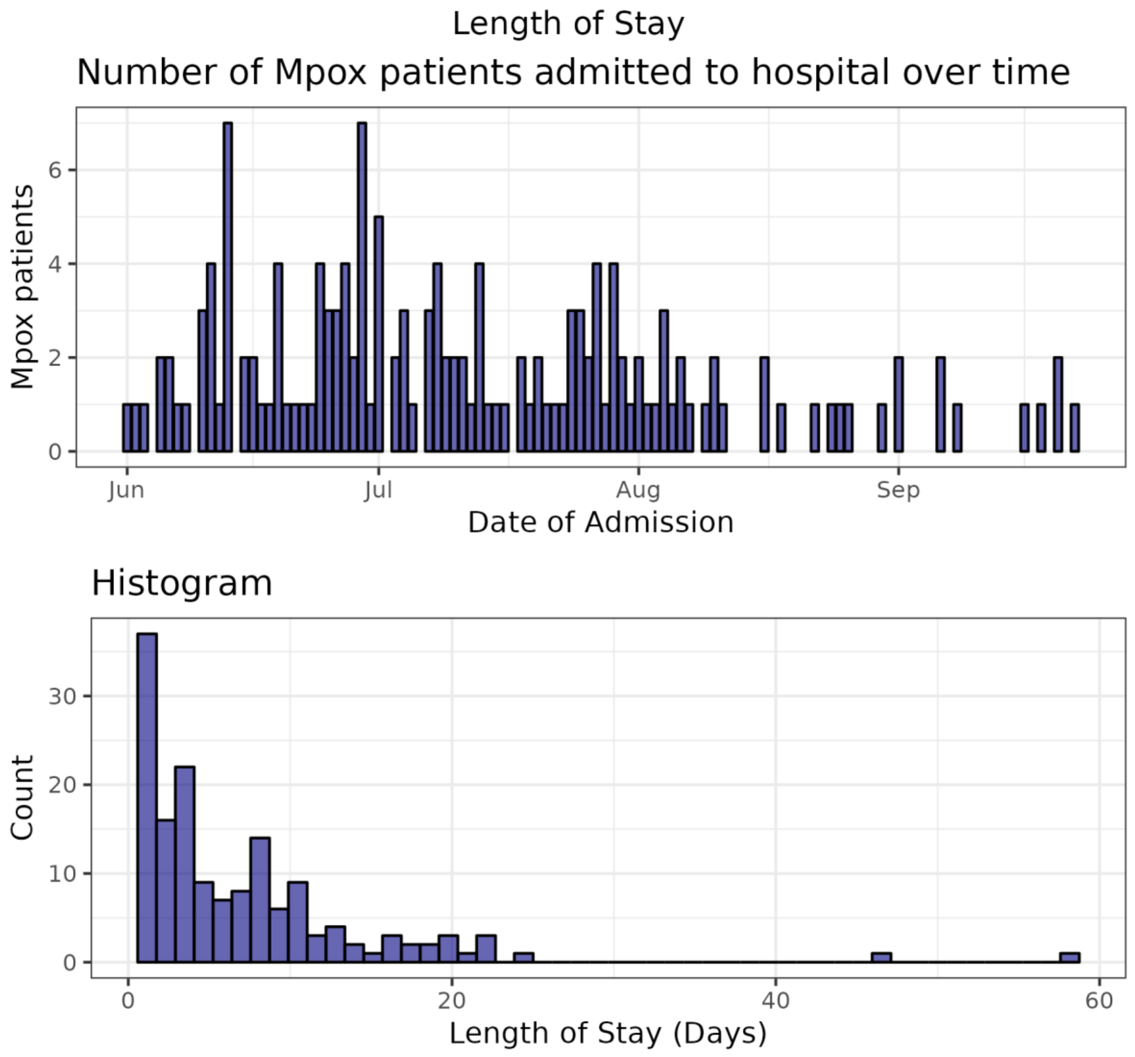

Supplementary Figure 4: The hospital admission dates of patients with an mpox infection and a histogram of the individual lengths of stay.

LOO

	Length of Stay		Infection to Hospitalisation		Infection to first positive test	
	RT		RT		RT	
	Loo	SE	Loo	SE	Loo	SE
**Lognormal**	910.4	29.0	788.0	18.1	578.5	19.2
**Weibull**	915.9	28.9	807.5	17.3	595.6	32.7
**Gamma**	915.1	28.8	796.0	18.2	577.5	23.7

Supplementary Table 1: Leave-one-out (LOO) cross validation scores for lognormal, Weibull and gamma distributions in each time delay model.

Infection to Hospital Admission

Infection to hospital admission						
N	Distribution	Mean	Standard Deviation	Shape/location	Scale	$\hat{R}$ (Mean)
118	Doubly interval censored adjusted for right truncation gamma	14.96 (13.64, 16.28)	7.02 (6.01, 8.20)	4.63 (3.45, 6.06)	0.31 (0.23, 0.40)	1.00
118	Doubly interval censored adjusted for right truncation Weibull	15.07 (13.72, 16.51)	7.50 (6.64, 8.54)	2.13 (1.83, 2.44)	17.00 (15.47, 18.63)	1.00

Supplementary Table 2: Summary statistics of the time from infection to hospital admissions for an mpox case, fit from data including 118 patients using gamma and Weibull distributions.

The cumulative distribution function estimates for the time from infection to hospital admissions					
Distribution	0.25 (CrI)	0.50 (CrI)	0.75 (CrI)	0.90 (CrI)	0.95 (CrI)
Doubly interval censored with right truncation lognormal	9.70 (8.59, 10.77)	13.33 (12.11, 14.58)	18.23 (16.72, 20.14)	24.29 (21.94, 27.44)	28.79 (25.62, 33.21)

Supplementary Table 3: The cumulative distribution function estimates for the time from infection to hospital admissions, fit to data from 118 cases using a lognormal distribution.

Infection to First Positive Test

Infection to first positive test						
N	Distribution	Mean	Standard Deviation	Shape/location	Scale	$\hat{R}$ (Mean)
86	Doubly interval censored adjusted for right truncation lognormal	15.62 (13.96, 17.62)	9.50 (7.48, 12.18)	2.59 (2.49, 2.69)	0.56 (0.48, 0.64)	1.00
86	Doubly interval censored adjusted for right truncation Weibull	15.24 (13.74, 16.86)	8.65 (7.60, 9.96)	1.83 (1.62, 2.04)	17.14 (15.43, 18.94)	1.00

Supplementary Table 4: Summary statistics of the time from infection to first positive test for an mpox case, fit to data from 86 cases using lognormal and Weibull distributions.

The cumulative distribution function estimates for the time from infection to first positive test					
Distribution	0.25 (CrI)	0.50 (CrI)	0.75 (CrI)	0.90 (CrI)	0.95 (CrI)
Doubly interval censored with right truncation gamma	9.35 (8.23, 10.51)	13.77 (12.36, 15.19)	19.35 (17.67, 21.40)	25.51 (23.17, 28.57)	29.70 (26.80, 33.62)

Supplementary Table 5: The cumulative distribution function estimates for the time from infection to first positive test, fit to data from 86 cases using a gamma distribution.

Length of Stay

Length of hospital stay						
N	Distribution	Mean	Standard Deviation	Shape/location	Scale	$\hat{R}$ (Mean)
155	Doubly interval censored adjusted for right truncation gamma	6.92 (6.09, 7.86)	6.76 (5.77, 7.87)	1.06 (0.88, 1.25)	0.15 (0.12, 0.19)	1.00
155	Doubly interval censored adjusted for right truncation Weibull	6.89 (6.05, 7.81)	6.90 (5.90, 8.12)	1.00 (0.90, 1.11)	6.87 (5.95, 7.86)	1.00

Supplementary Table 6: Summary statistics of the time of the length of stay in hospital for an mpox patient, fit to data from 155 patients using a lognormal distribution.

The cumulative distribution function estimates for the length of hospital stay					
Distribution	0.25 (CrI)	0.50 (CrI)	0.75 (CrI)	0.90 (CrI)	0.95 (CrI)
Doubly interval censored with right truncation lognormal	1.97 (1.67, 2.33)	4.03 (3.52, 4.60)	8.21 (7.17, 9.49)	15.55 (13.13, 18.58)	22.84 (18.89, 28.12)

Supplementary Table 7: The cumulative distribution function estimates for the length of hospital stay, fit to data from 155 patients using a lognormal distribution.

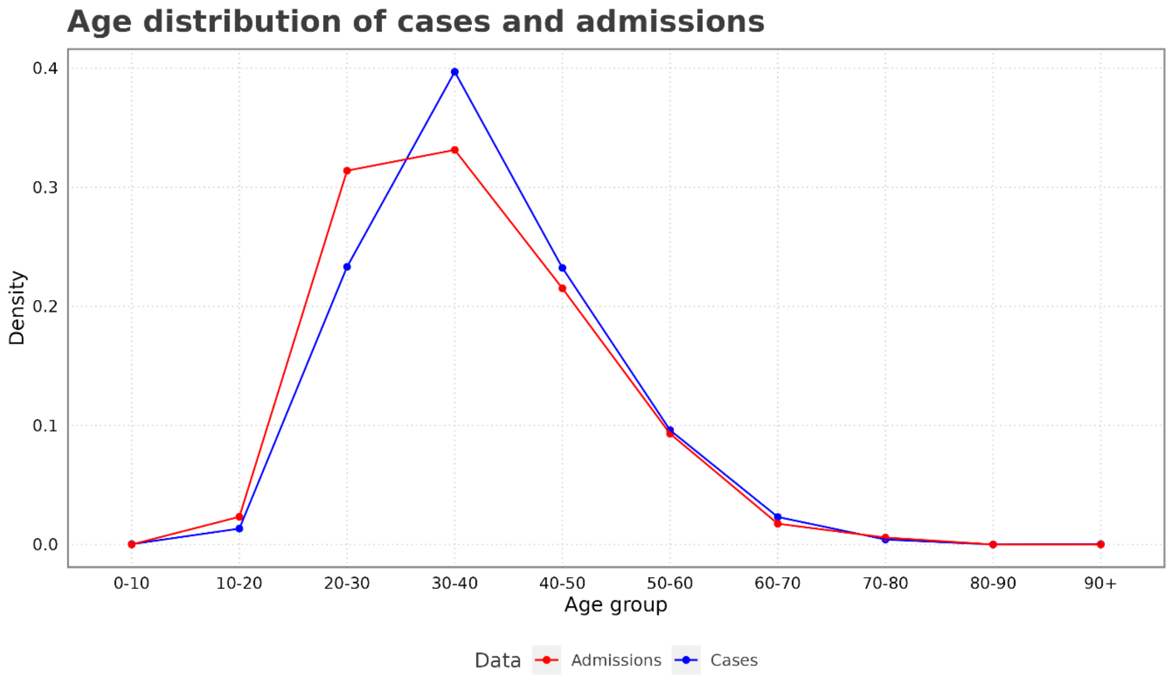

Supplementary Figure 5 A density plot of confirmed mpox RT-PCR cases and hospital admissions by age group.

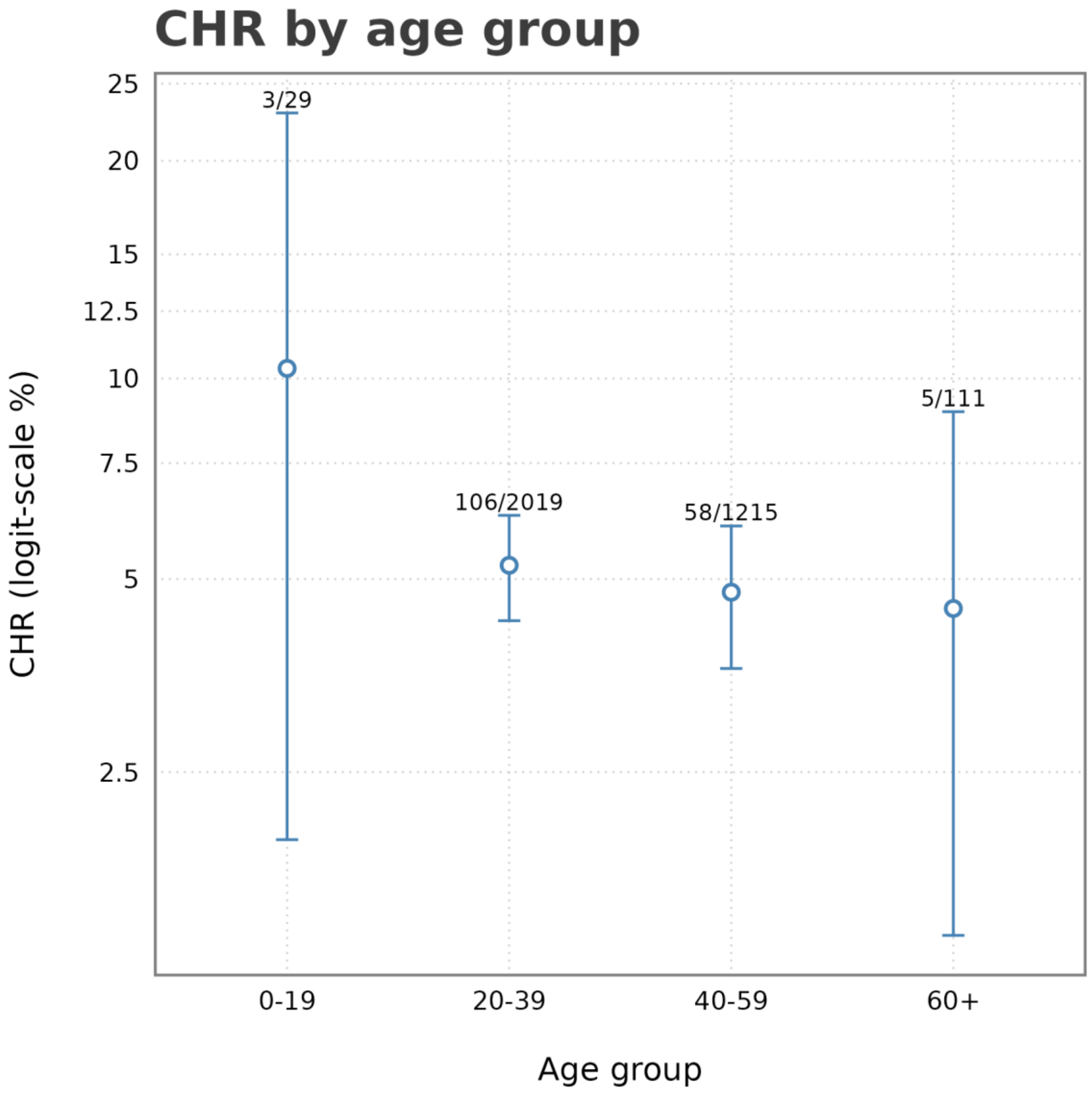

Supplementary Figure 6 The age-specific case hospitalisation risks with 95% binomial credible intervals.

Case hospitalisation risk	Posterior Estimate (%)
Index cases	8.47 (95% CI: 7.08, 9.97)
Secondary cases	4.54 (95% CI: 3.81, 5.25)
Untraced cases	4.77 (95% CI: 4.17, 5.43)

Supplementary Table 8: Posterior estimates of the case hospitalisation risks, when assuming that untraced cases are a mixture of index and secondary cases.

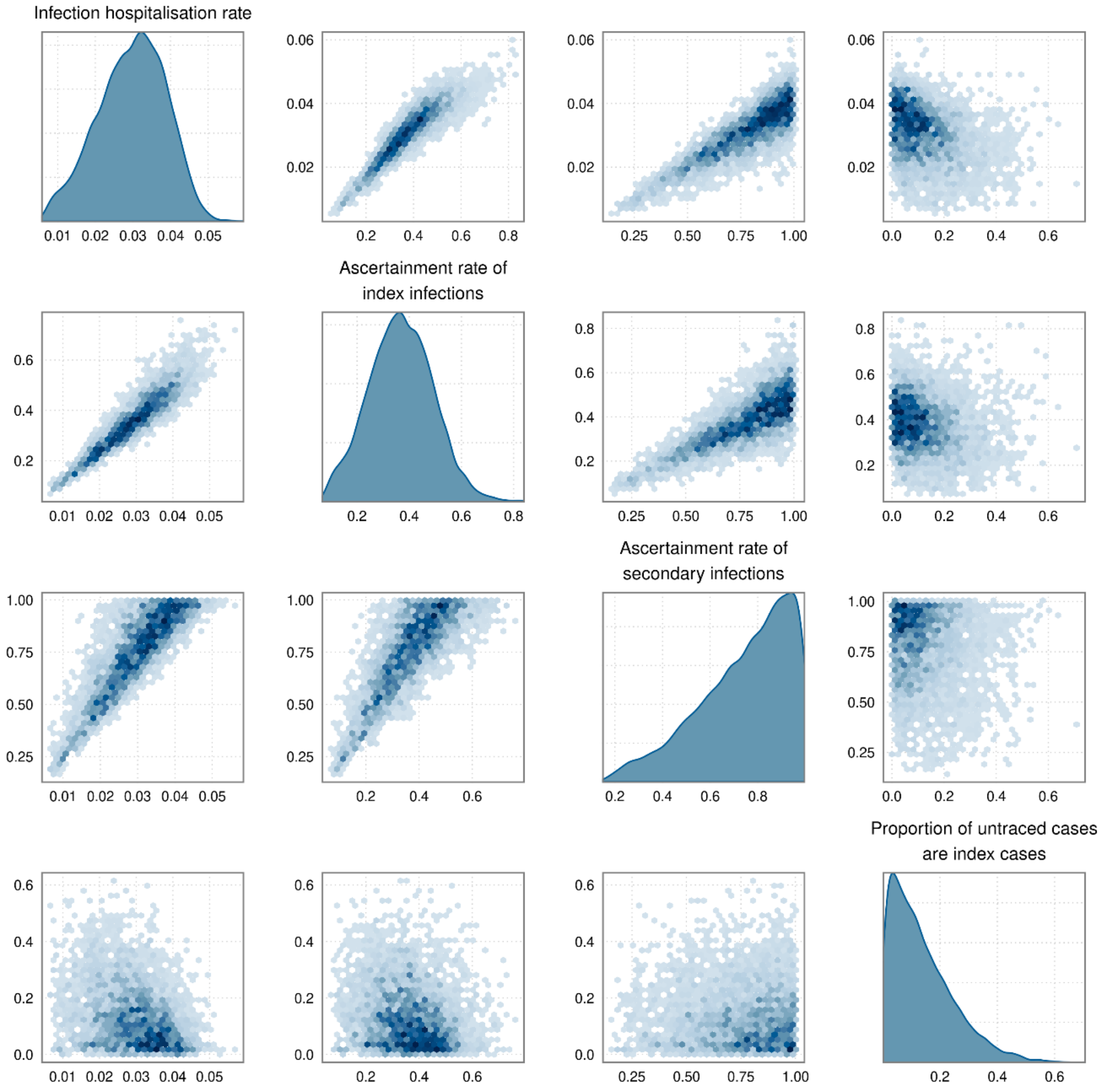

Supplementary Figure 7: The infection ascertainment prior distribution for key parameters of interest. These parameters are (in order), the infection hospitalisation risk, the probability of ascertaining non-hospitalised index/secondary infections, and the proportion of untraced cases that are estimated to be index cases. The diagonal plots are estimates of the posterior density, and the off-diagonal estimates are pairwise posterior density estimates.

Parameter	Symbol	Posterior Estimate (%)
Hospitalisation risk	p_h	4.13 (95% CI: 3.04, 5.02)
Probability of ascertaining an index *infection*	$\alpha_p(1-p_h)+p_h$	49.13 (95% CI: 34.80, 63.95)
Probability of ascertaining a secondary infection	$\alpha_s(1-p_h)+p_h$	90.84 (95% CI: 70.18, 99.74)
Proportion of untraced infections that were index *infections*	$\mathrm{P(U=p)}$	10.08 (95% CI: 0.31, 32.80)

Supplementary Table 9: Posterior estimates of our key quantities of interest.

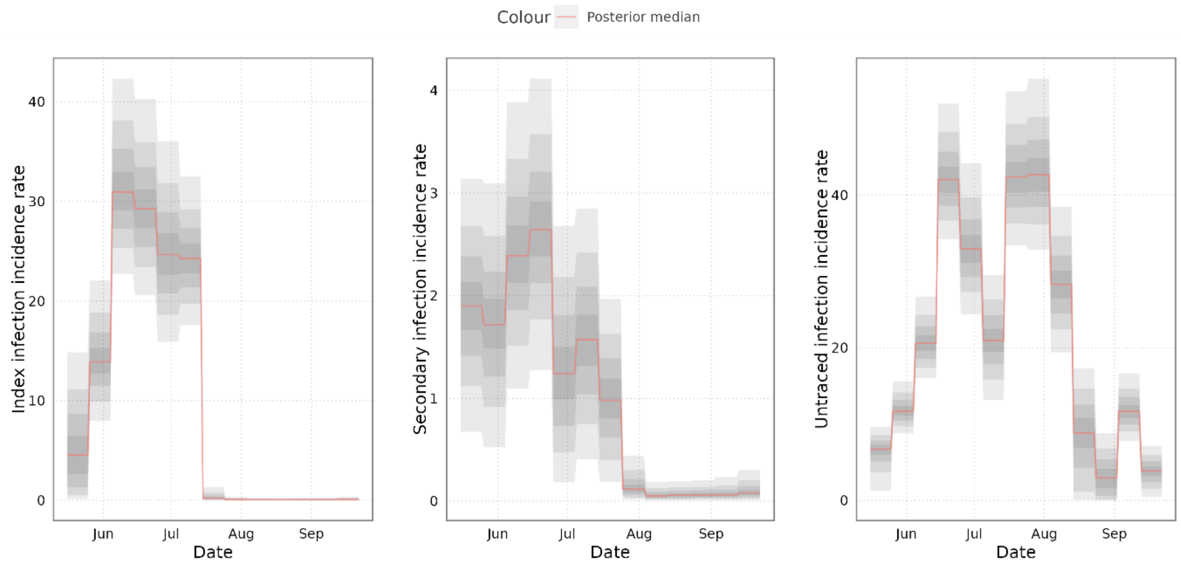

Supplementary Figure 8: Expected incidence rate over time for the three subpopulations. Each panel displays the estimate of the incidence rate over time for a different subpopulation. The central red line displays the posterior median, and the grey shaded areas display the uncertainty in the estimate, with the lightest region displaying the 90% credible intervals. We estimate the incidence for 10-day intervals, which results in step changes in the incidence.

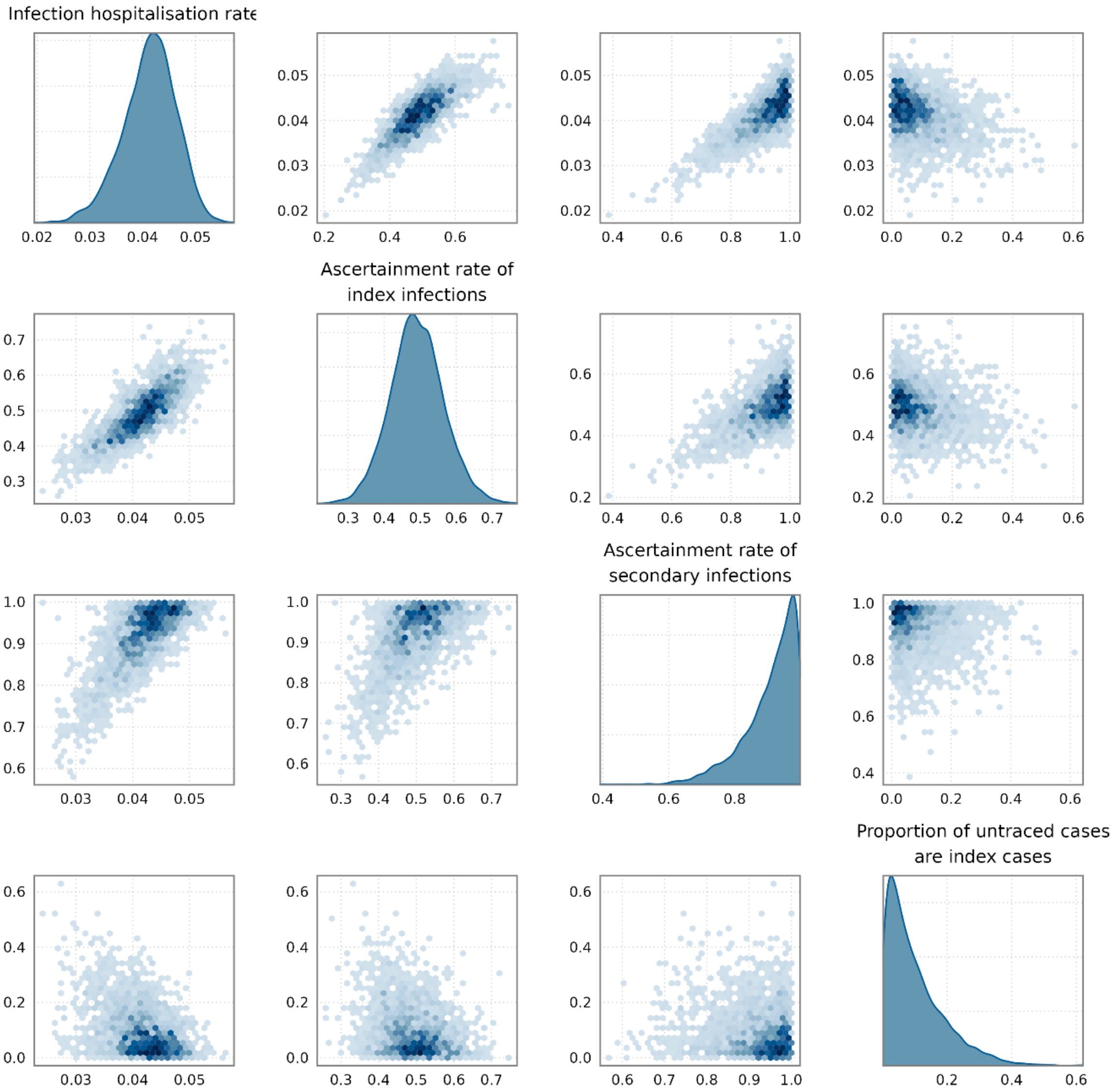

Supplementary Figure 9: Visualisation of the posterior for key parameters of interest from fitting the incidence rate model. These parameters are (in order), the infection hospitalisation risk, the probability of ascertaining index/secondary cases, and the proportion of untraced cases that are estimated to be index cases. The diagonal plots are estimates of the posterior density, and the off-diagonal estimates are pairwise posterior density estimates.

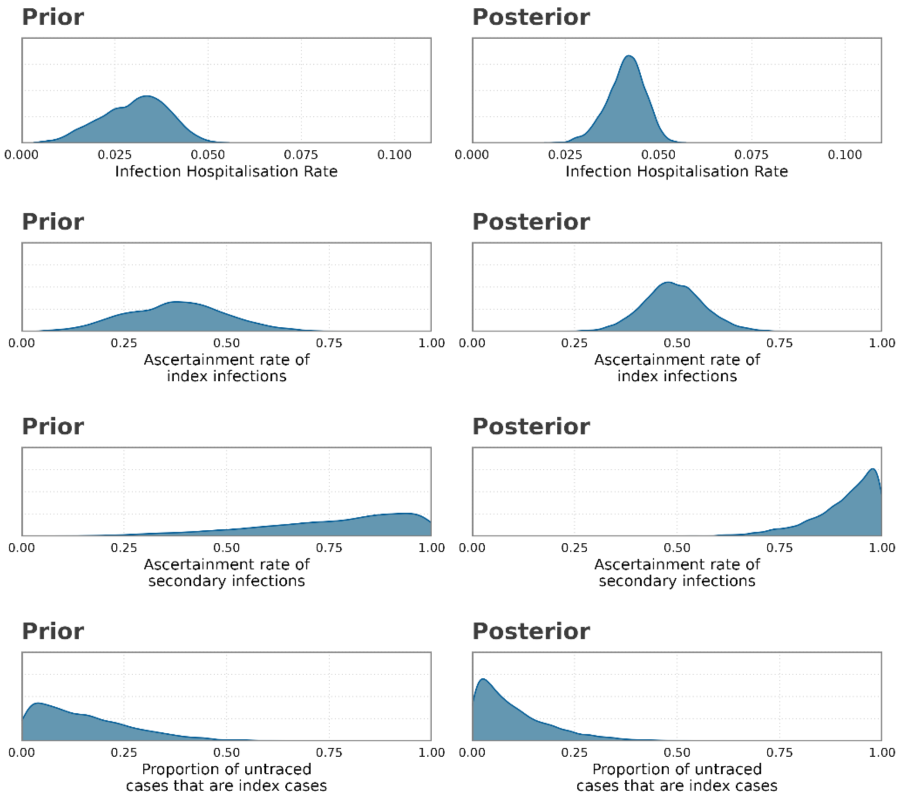

Supplementary Figure 10: A comparison of the infection ascertainment prior distribution against the full model posterior obtained by fitting incidence over time and including untraced cases.

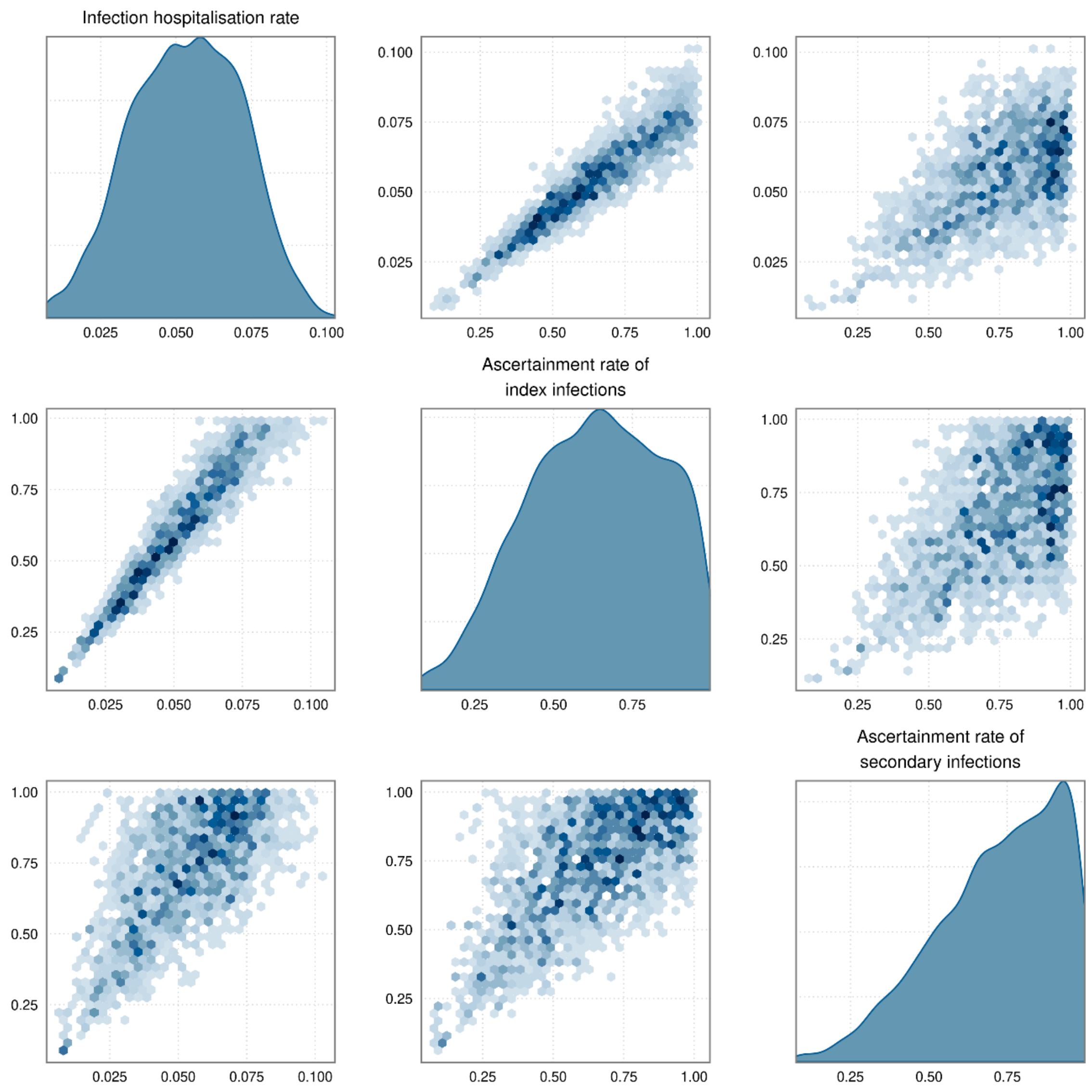

Supplementary Figure 11: The infection ascertainment prior distribution for key parameters of interest, when the untraced group is omitted. These parameters are (in order), the infection hospitalisation risk, the probability of ascertaining non-hospitalised index/secondary infections, and the proportion of untraced cases that are estimated to be index cases. The diagonal plots are estimates of the posterior density, and the off-diagonal estimates are pairwise posterior densities estimates.

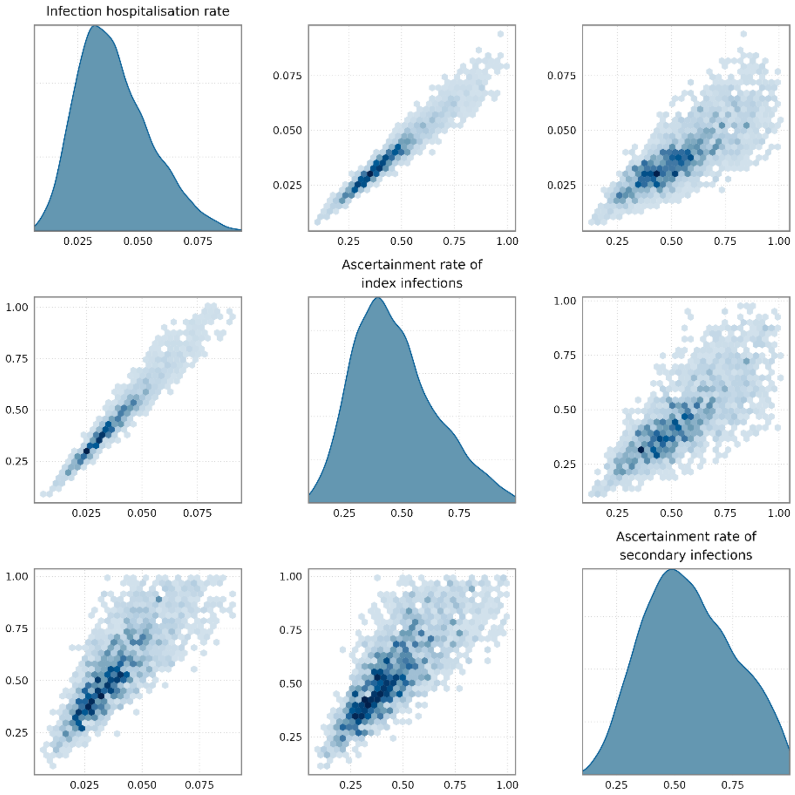

Supplementary Figure 12: Visualisation of the posterior for key parameters of interest obtained from fitting the incidence rate model with the untraced group omitted. These parameters are (in order), the infection hospitalisation risk, the probability of ascertaining index/secondary infections, and the proportion of untraced cases that are estimated to be index infections. The diagonal plots are estimates of the posterior density, and the off-diagonal plots are estimates of the pairwise posterior density.

Parameter	Symbol	Posterior Estimate (%)
Probability of hospitalisation	p_h	3.99 (95% CI: 1.62, 7.31)
Probability of ascertaining index infection	$\alpha_p(1 - p_h) + p_h$	46.97 (95% CI: 18.65, 86.96)
Probability of ascertaining a secondary infection	$\alpha_s(1 - p_h) + p_h$	56.48 (95% CI: 23.32, 94.06)

Supplementary Table 10: Posterior estimates of our key quantities of interest, when the the untraced group is omitted.

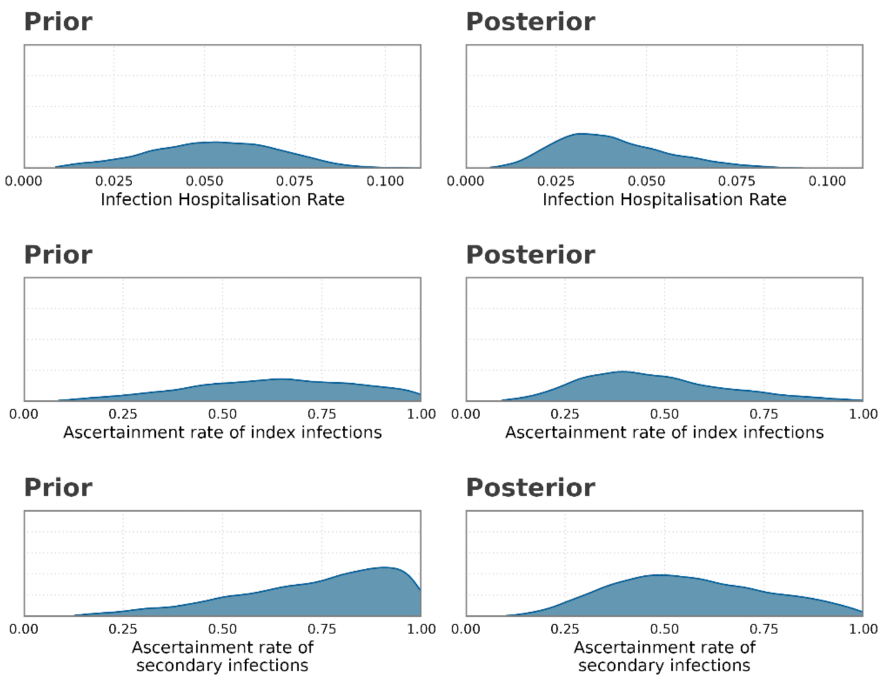

Supplementary Figure 13: Comparison of prior density of the infection ascertainment bias prior when the untraced group is omitted, against the posterior density obtained when fitting the incidence rate model when the untraced group is omitted.

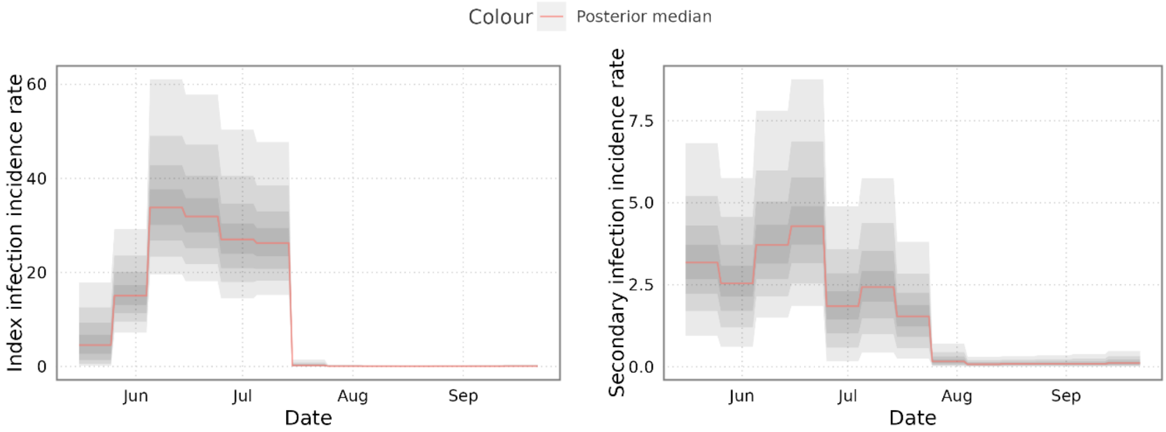

Supplementary Figure 14: Expected incidence rate over time for the two index and secondary subpopulations, when the untraced group is omitted. Each panel displays the estimate of the incidence rate over time for a different subpopulation. The central red line displays the posterior median, and the grey shaded areas display the un uncertainty in the estimate, with the lightest region displaying the 90% credible intervals. We estimate the incidence for 10-day intervals, which results in step changes in the incidence.

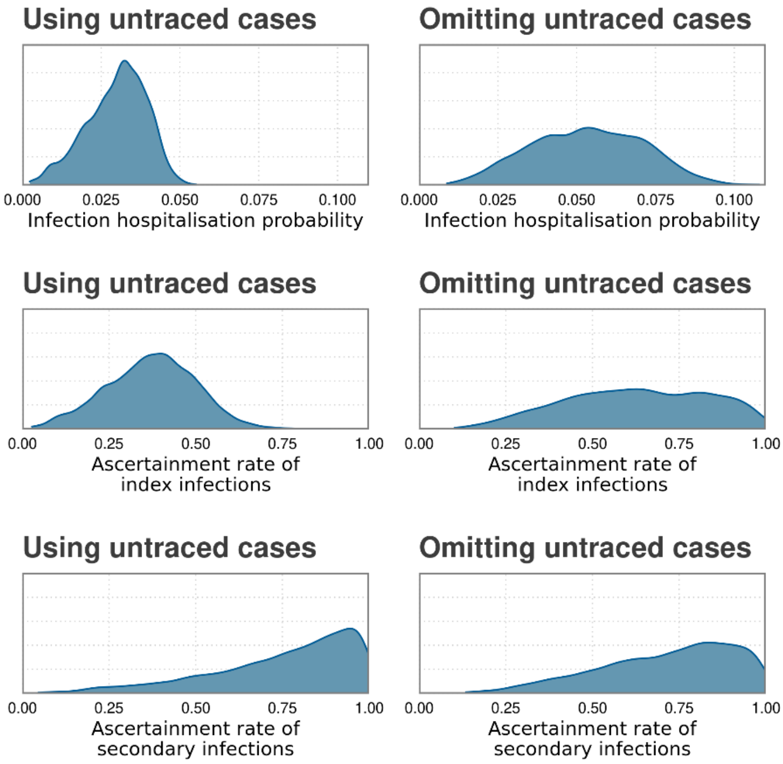

Supplementary Figure 15: Comparison of prior densities for key parameters when untraced cases are included (left) or excluded (right).

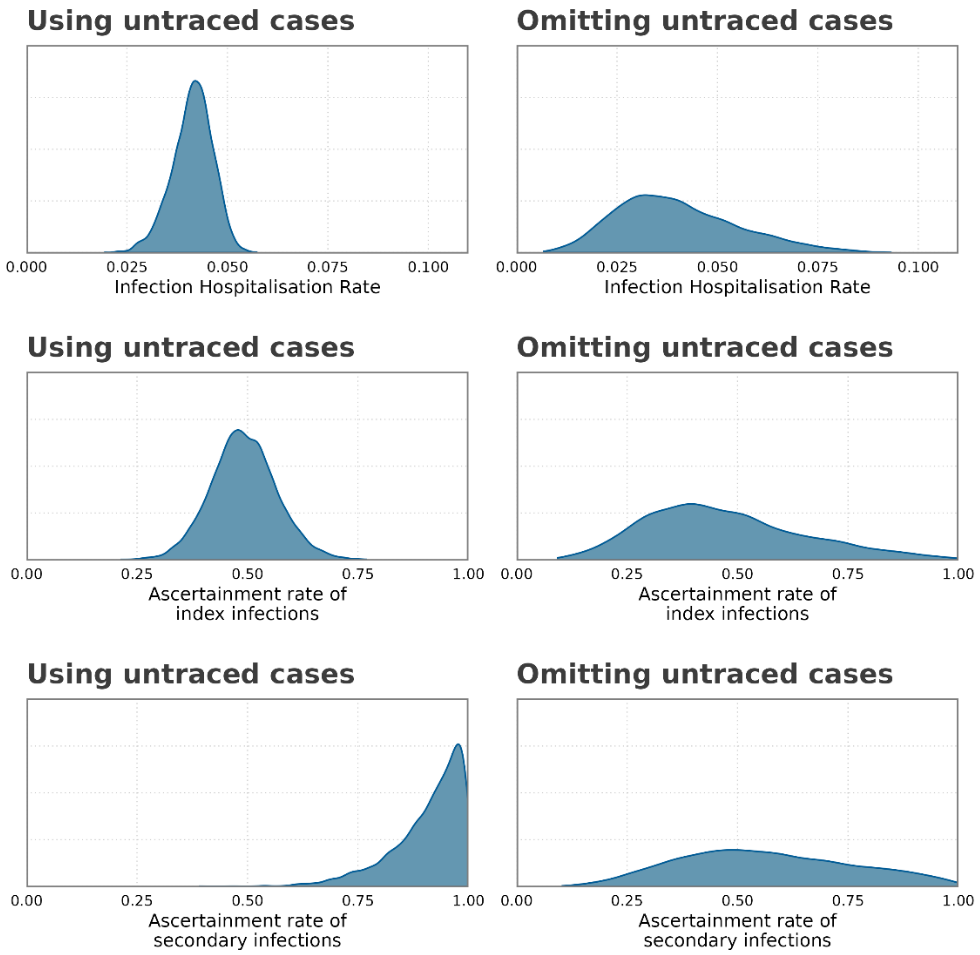

Supplementary Figure 16: Comparison of posterior densities for key parameters of interest, when the model is fit with untraced cases included (left), and when the model is fit with untraced cases excluded (right).

	No of cases	Mean age (SD) years	% Male (Count/Total Count)	% GBMSM (Count/Total Count)
All mpox cases during the study period	3,375	37.9 (10.2)	99.2 (3347/3375)	95.5 (1177/1232)
All mpox cases with a hospital episode	824	37.3 (10.3)	98.8 (814/824)	94.3 (348/369)
Mpox cases used to calculate length of stay	155	36.3 (11.2)	96.8 (150/155)	92.2 (71/77)
Mpox cases used to calculate symptom onset to hospitalisation	110	36.0 (11.3)	95.5 (105/110)	90.6 (58/64)
Mpox cases used to calculate exposure to hospitalisation	8	32.9 (5.1)	100 (8/8)	100 (8/8)

Supplementary Table 11: The number of cases, the mean age of each study sample, the proportion of cases that were male and that identified as GBMSM.

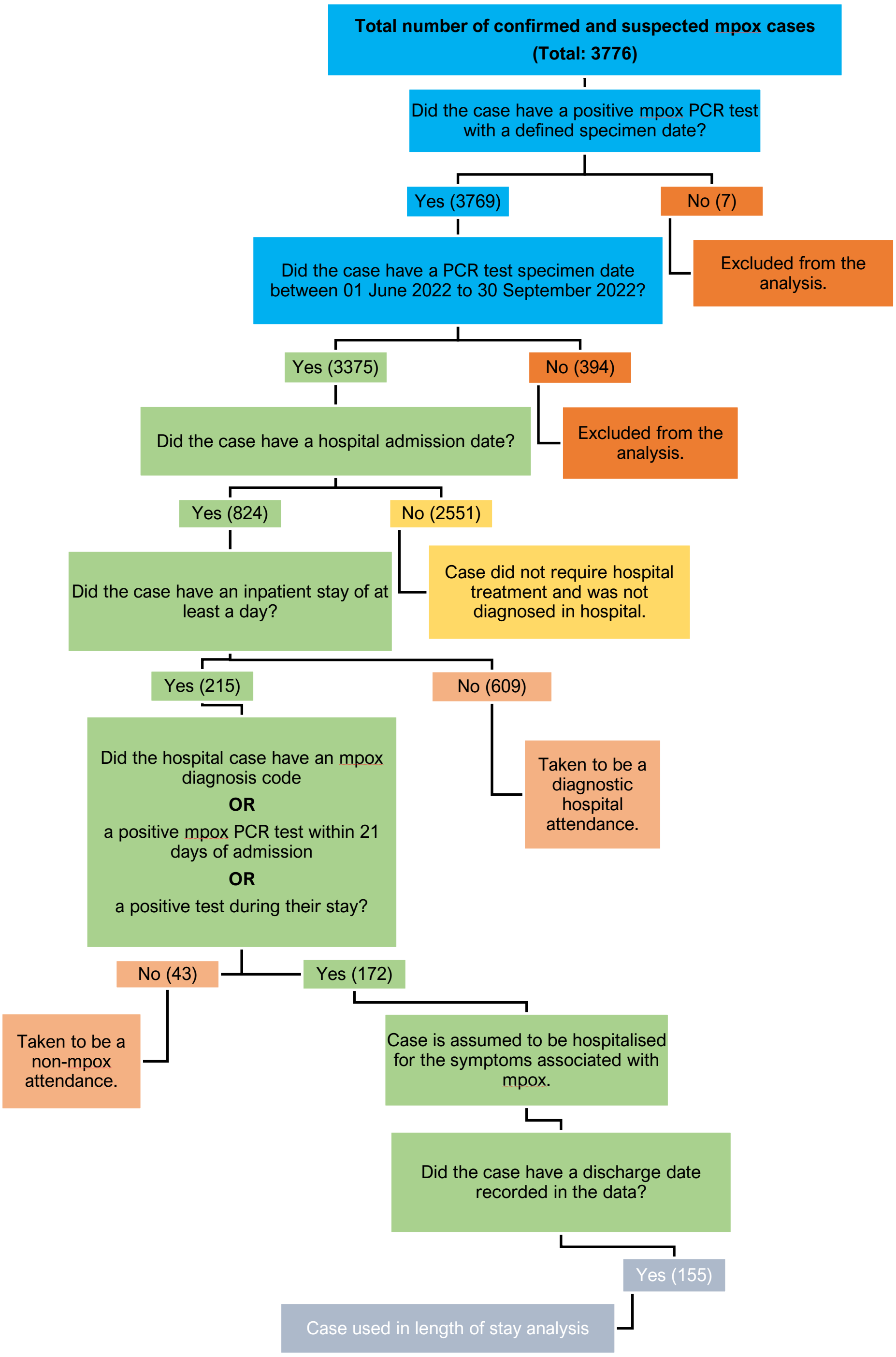

Supplementary Figure 17: A flow chart of the mpox data inclusion criteria.
