# Supplementary material for: Understanding the infection severity and epidemiological characteristics of mpox in the UK
Source: Nat Commun. 2024 Mar 11;15:2199. doi: 10.1038/s41467-024-45110-8 (PMC10928097; doi:10.1038/s41467-024-45110-8)
Supplement: Supplementary file 3 — Description of Additional Supplementary Files [file 41467_2024_45110_MOESM3_ESM.pdf]

Description of Additional Supplementary Files

File Name: Supplementary Code
Description: The Stan code to model the infection hospitalisation risk and the doubly interval censored model adjusted for right truncation.
