# Supplementary material for: Understanding the infection severity and epidemiological characteristics of mpox in the UK
Source: Nat Commun. 2024 Mar 11;15:2199. doi: 10.1038/s41467-024-45110-8 (PMC10928097; doi:10.1038/s41467-024-45110-8)
Supplement: Supplementary file 5 — Reporting Summary [file 41467_2024_45110_MOESM5_ESM.pdf]

nature portfolio

Corresponding author(s): Thomas Ward

Last updated by author(s): Nov 10, 2023

Reporting Summary

Nature Portfolio wishes to improve the reproducibility of the work that we publish. This form provides structure for consistency and transparency in reporting. For further information on Nature Portfolio policies, see our Editorial Policies and the Editorial Policy Checklist.

Statistics

For all statistical analyses, confirm that the following items are present in the figure legend, table legend, main text, or Methods section.

n/a	Confirmed	
☐	☒	The exact sample size (*n*) for each experimental group/condition, given as a discrete number and unit of measurement
☐	☒	A statement on whether measurements were taken from distinct samples or whether the same sample was measured repeatedly
☒	☐	The statistical test(s) used AND whether they are one- or two-sided<br>*Only common tests should be described solely by name; describe more complex techniques in the Methods section.*
☐	☒	A description of all covariates tested
☐	☒	A description of any assumptions or corrections, such as tests of normality and adjustment for multiple comparisons
☐	☒	A full description of the statistical parameters including central tendency (e.g. means) or other basic estimates (e.g. regression coefficient) AND variation (e.g. standard deviation) or associated estimates of uncertainty (e.g. confidence intervals)
☒	☐	For null hypothesis testing, the test statistic (e.g. *F*, *t*, *r*) with confidence intervals, effect sizes, degrees of freedom and *P* value noted<br>*Give P values as exact values whenever suitable.*
☐	☒	For Bayesian analysis, information on the choice of priors and Markov chain Monte Carlo settings
☐	☒	For hierarchical and complex designs, identification of the appropriate level for tests and full reporting of outcomes
☒	☐	Estimates of effect sizes (e.g. Cohen's *d*, Pearson's *r*), indicating how they were calculated

Our web collection on statistics for biologists contains articles on many of the points above.

Software and code

Policy information about availability of computer code

Data collection	AWS, R version 4.3.2, SQL RTM version is 16.0.1000.6
Data analysis	R version 4.3.2, cmdstanr 0.6.1, SQL RTM version is 16.0.1000.6, AWS

For manuscripts utilizing custom algorithms or software that are central to the research but not yet described in published literature, software must be made available to editors and reviewers. We strongly encourage code deposition in a community repository (e.g. GitHub). See the Nature Portfolio guidelines for submitting code & software for further information.

Data

Policy information about availability of data

All manuscripts must include a data availability statement. This statement should provide the following information, where applicable:

- Accession codes, unique identifiers, or web links for publicly available datasets
- A description of any restrictions on data availability
- For clinical datasets or third party data, please ensure that the statement adheres to our policy

UKHSA operates a robust governance process for applying to access protected data that considers:

• the benefits and risks of how the data will be used
• compliance with policy, regulatory and ethical obligations

• data minimisation
• how the confidentiality, integrity, and availability will be maintained
• retention, archival, and disposal requirements
• best practice for protecting data, including the application of ‘privacy by design and by default’, emerging privacy conserving technologies and contractual controls
Access to protected data is always strictly controlled using legally binding data sharing contracts.
UKHSA welcomes data applications from organisations looking to use protected data for public health purposes.
To request an application pack or discuss a request for UKHSA data you would like to submit, contact DataAccess@ukhsa.gov.uk.

Research involving human participants, their data, or biological material

Policy information about studies with human participants or human data. See also policy information about sex, gender (identity/presentation), and sexual orientation and race, ethnicity and racism.

Reporting on sex and gender	Analysis of the hospitalisation risk differences between sex has been included.
Reporting on race, ethnicity, or other socially relevant groupings	We have included a description of the data that includes self reported sexual behaviour differences, which is described in the data section of the methods.
Population characteristics	The characteristics of the population included for analysis has been included in the data section of the methods.<br><br>Data was extracted on the 26th October 2022, at which time 3,776 people had tested positive for the monkeypox virus in the UK, 3,375 of which had specimen date within the study period, and 172 of those had an associated hospital episode of 1 day or longer. Length of stay was calculated from the date of admission to the date of discharge and patients were excluded if the discharge date was missing. This is due to the nature of the hospital admissions data, where records are only reported after discharge, so the discharge dates for these patients will be incorrectly missing rather than representing patients still in hospital. This resulted in 155 patients being suitable for the length of stay analysis. Supplementary Figure 17 shows a flowchart with the number of cases excluded at each stage.<br><br>To calculate the time from infection to hospitalisation, patients that reported a symptom onset date after hospitalisation were excluded (since this likely corresponded to incorrect symptom questionnaire data) and for those that had multiple admissions the earliest admission date was used. The symptom onset date was identified through contact tracing conducted by UKHSA health protection teams and questionnaires completed by the cases (via the question “On what date did your illness begin?”). This definition of symptom onset describes the date that an individual first noticed their symptoms; though the true date of symptom onset could have been earlier but not detected. Of the 3,776 monkey pox cases identified, 2,360 had symptom onset information, of which 110 had a hospital admission. Analysis was also conducted to measure the time from infection to hospitalisation for patients that had exposure dates reported. Exposure date was identified through cases which had completed a questionnaire and answered the questions “In the 21 days (3 weeks) before first symptom onset did you have contact with anyone with suspected or confirmed monkeypox infection?” and “Date of last contact with case”. 92 patients of the 3,776 reported an exposure date, 8 of which had a hospital admission. Then of the 92 patients with exposure date, 86 had a reliable specimen collection date, and were used for estimating the delay from infection date to first positive test.<br><br>Mean age (and SD) of all mpox cases during the study period - 37.9 (10.2). Please see (Supplementary Table 11) for full characteristics.
Recruitment	Cases of the monkeypox virus were monitored by the UKHSA using testing data from affiliated laboratories and NHS laboratories, contact tracing, and case questionnaires (collected by UKHSA health protection teams). A confirmed case is an individual with a positive PCR test result for the monkeypox virus, and a highly probable case as an individual with a positive PCR test result for orthopoxvirus. As of 25th July 2022, both definitions were recognised in the UK to represent a case of mpox.<br>Hospital episode statistics for inpatients were obtained from the NHS Digital Secondary Uses Services data set and A&E attendances were obtained from the Emergency Care Data Set, both datasets contain clinical, patient, administrative and geographic information about patient admissions. A&E attendances and inpatient records were extracted, and hospital episodes linked by an NHS identifier to a positive test result. An admission for mpox is defined as a patient having one of the following:<br>• A monkeypox diagnosis code (B04).<br>• A positive PCR for the monkeypox virus within 21 days after admission to hospital.<br>• Tested positive for the monkeypox virus during a hospital admission.
Ethics oversight	This study was conducted for the purpose of informing the outbreak response to the monkeypox pandemic. Work was undertaken in line with national and UKHSA data regulations.

Note that full information on the approval of the study protocol must also be provided in the manuscript.

Field-specific reporting

Please select the one below that is the best fit for your research. If you are not sure, read the appropriate sections before making your selection.

☒ Life sciences ☐ Behavioural & social sciences ☐ Ecological, evolutionary & environmental sciences

For a reference copy of the document with all sections, see nature.com/documents/nr-reporting-summary-flat.pdf

Life sciences study design

All studies must disclose on these points even when the disclosure is negative.

Sample size

Data was extracted on the 26th October 2022, at which time 3,776 people had tested positive for the monkeypox virus in the UK, 3,375 of which had specimen date within the study period, and 172 of those had an associated hospital episode of 1 day or longer. Length of stay was calculated from the date of admission to the date of discharge and patients were excluded if the discharge date was missing. This is due to the nature of the hospital admissions data, where records are only reported after discharge, so the discharge dates for these patients will be incorrectly missing rather than representing patients still in hospital. This resulted in 155 patients being suitable for the length of stay analysis. Supplementary Figure 17 shows a flowchart with the number of cases excluded at each stage.

To calculate the time from infection to hospitalisation, patients that reported a symptom onset date after hospitalisation were excluded (since this likely corresponded to incorrect symptom questionnaire data) and for those that had multiple admissions the earliest admission date was used. The symptom onset date was identified through contact tracing conducted by UKHSA health protection teams and questionnaires completed by the cases (via the question "On what date did your illness begin?"). This definition of symptom onset describes the date that an individual first noticed their symptoms; though the true date of symptom onset could have been earlier but not detected. Of the 3,776 monkey pox cases identified, 2,360 had symptom onset information, of which 110 had a hospital admission. Analysis was also conducted to measure the time from infection to hospitalisation for patients that had exposure dates reported. Exposure date was identified through cases which had completed a questionnaire and answered the questions "In the 21 days (3 weeks) before first symptom onset did you have contact with anyone with suspected or confirmed monkeypox infection?" and "Date of last contact with case". 92 patients of the 3,776 reported an exposure date, 8 of which had a hospital admission. Then of the 92 patients with exposure date, 86 had a reliable specimen collection date, and were used for estimating the delay from infection date to first positive test.

In Supplementary Table 11 we report for each sample the sample size, the mean age, the proportion by sex, and proportion of cases reported as GBMSM. Supplementary Figures 1-4 show histograms of the time delay data for symptom onset to hospital admission, exposure to hospital admission, exposure to first positive test, and length of stay, respectively.

Data exclusions

An admission for mpox is defined as a patient having one of the following:
• A monkeypox diagnosis code (B04).
• A positive PCR for the monkeypox virus within 21 days after admission to hospital.
• Tested positive for the monkeypox virus during a hospital admission.

Therefore, individuals that did not fulfill this criteria would not be included as a hospital admission for mpox.

A full diagram of the exclusion criteria is provided in the Supplementary Materials of the paper.

Replication

All statistical models were repeatedly tested to assess prior distributions and sensitivity analysis was conducted of the model structures, which is included in the supplementary section.

Randomization

All available data were included in the analysis and we did not have experimental groupings.

Blinding

N/A

Reporting for specific materials, systems and methods

We require information from authors about some types of materials, experimental systems and methods used in many studies. Here, indicate whether each material, system or method listed is relevant to your study. If you are not sure if a list item applies to your research, read the appropriate section before selecting a response.

Materials & experimental systems

n/a	Involved in the study	
☒	☐	Antibodies
☒	☐	Eukaryotic cell lines
☒	☐	Palaeontology and archaeology
☒	☐	Animals and other organisms
☒	☐	Clinical data
☒	☐	Dual use research of concern
☒	☐	Plants

Methods

n/a	Involved in the study	
☒	☐	ChIP-seq
☒	☐	Flow cytometry
☒	☐	MRI-based neuroimaging
